# Bee Brood as a Food for Human Consumption: An Integrative Review of Phytochemical and Nutritional Composition

**DOI:** 10.3390/insects16080796

**Published:** 2025-07-31

**Authors:** Raquel P. F. Guiné, Sofia G. Florença, Maria João Barroca, Cristina A. Costa

**Affiliations:** 1 CERNAS-IPV Research Centre, Polytechnic University of Viseu, Campus Politécnico, 3504-510 Viseu, Portugal; sofiaflorenca@outlook.com (S.G.F.); amarocosta@esav.ipv.pt (C.A.C.); 2 ESAC—Agriculture School of Coimbra, IPC—Polytechnic University of Coimbra, Bencanta, 3045-601 Coimbra, Portugal; mjbarroca@esac.pt

**Keywords:** bee brood, *A. mellifera*, nutritional value, chemical composition

## Abstract

The utilisation of edible insects for human nutrition is a long-standing practice in many parts of the globe, and is being gradually introduced into countries without an entomophagic tradition as well. These unconventional sources of protein of animal origin have arisen as a sustainable alternative to other animal protein sources, such as meat. This review intends to present the compilation of data in the scientific literature on the chemical composition and nutritional value of the bee brood of *A. mellifera* species and subspecies as edible foods. For this, a comprehensive search of the scientific literature was carried out using the databases ScienceDirect, Scopus, Pub-Med, BOn, and SciELO. Appropriate keywords were used for the search to reach the research works that addressed the topics of the review. The results showed that bee brood has considerable quantities of protein, fat and carbohydrates. The most abundant amino acids are leucine and lysine (these two being essential amino acids) and aspartic acid, glutamic acid, and proline (these three being non-essential amino acids). As for the fatty acids, bee broods contain approximately equal fractions of saturated and monounsaturated fatty acids, while the polyunsaturated fatty acids are negligible. The dietary minerals present in higher quantities are potassium, phosphorus, and magnesium, and the most abundant vitamins are vitamin C and niacin; choline is also present, although it is not a true vitamin. Although bee brood from *A. mellifera* has potential for human consumption as a nutrient-rich food, there are still many aspects that need to be further studied in the future, such as safety and hazards linked to possible regular consumption.

## Simple Summary

Edible insects are part of the traditional culinary practices for many people worldwide. These foods are important sources of macro and micro nutrients and also bioactive compounds that play significant roles in the human body when ingested as part of the daily diet. This review article aims to compile scientific data reporting the chemical composition and nutritional and biological value of bee brood. The results confirmed that bee brood has considerable quantities of protein, fat, carbohydrates, minerals, and vitamins, which are important nutrients. Besides, it contains important amino acids and fatty acids, including unsaturated fatty acids, which are considered healthy fats. For these reasons, bee brood is confirmed as a nutritive food. However, some aspects still need further assessment, such as safety concerns and possible hazards linked to regular consumption.

## 1. Introduction

Providing food security for the increasing world population is a challenge for the present and future generations. By 2050, it is expected that the world population will reach 10 billion, and feeding the increasing number of people on the planet is a priority [1].

Edible insects have been suggested as a way to provide protein-rich foods of animal origin while being considerably more sustainable than other foods of animal origin [2]. Edible insects provide a cheaper protein source in comparison to meat and require smaller quantities of natural resources to produce the same amount of protein as other domesticated animals [3]. Producing 1 kg of insects requires less than 0.1% of the water needed to produce 1 kg of beef (only 4 litres against 15.4 tons of water) [4,5], and water is becoming more and more scarce and valuable over the years. As for feed, only 8% of the feed needed for cows to produce 1 kg of beef is needed to produce the same amount of insects [6]. Compared with other animals used for human food, insects require much less feed, land, and water than cows, pigs, and chickens [6]. However, their utilisation as human food is not well accepted globally.

Humans have consumed insects for a long time in some parts of the world, where entomophagy is a traditional practice. However, in Western societies, insects are not always readily accepted due to unfamiliarity, suspicion, and even neophobia. The work by Florença et al. [7] compared determinants for acceptance of edible insects among insect-eating countries and Western countries and found that in countries with entomophagy tradition, the motivations for consuming insects relate to sensory attributes, availability, affordability, and preferences, while in Western countries the determinants for eating insects are nutritional value and sustainability, and those for not eating them are neophobia and disgust. However, it was also observed that Western consumers are more receptive to consuming food products containing insects instead of whole insects. Some larvae from insects that are consumed by humans include those from palm weevil, flower beetle, mealworm beetle, and honey bee [1,3,8,9,10].

Honey bees (*A. mellifera*) have been nurtured by humans for millennia across the world. The life cycle of honey bees encompasses four principal stages, starting with the egg, then developing the larvae, progressing to the pupa and finally reaching the adult phase [3]. In these different stages, the physiology of the larvae changes, and so it happens with the chemical composition and elements of nutritional value, such as the protein and its amino acids, the fat and the corresponding fatty acids, minerals, or vitamins.

The eggs, larvae, and pupae of honeybees, which develop within the beehive, are considered bee products. Apilarnil, in particular, is composed of drone bee larvae (3–11 days after hatching), also known as drone brood homogenate (DBH) [11,12]. It is obtained through a mechanical homogenisation process that produces raw apilarnil—a milky substance with a creamy texture and a colour range from white to yellow.

Although drone larvae are often treated as a waste product by beekeepers, they are a rich source of bioactive compounds. These include proteins (9–12%), enzymes, carbohydrates (6–10%), lipids (5–8%), steroid hormones, vitamins, minerals, essential amino acids, and other biologically active substances. These components contribute to a variety of pharmacological effects, such as antioxidant [13], neuroprotective, anti-apoptotic, anti-inflammatory, and androgenic activities. They also serve as an effective apitherapeutic agent in wound healing and exhibit protective potential against brain damage [11,14,15,16,17,18]. This product has been compared with royal jelly, which is a milky secretion produced by worker honeybees for the purpose of feeding larvae and adult bee queens [17]. However, the antioxidant potential of drone brood is reported to be greater than that of royal jelly [13].

One advantage of honeybee larvae is the positive connotation to the honeybees, their familiarity with human, their capability of giving us honey, and their role as pollinators. All these positive perceptions are allies to the introduction of honeybee brood into human consumption in Western societies. Nevertheless, it is important to inform consumers that the consumption of drone brood does not imply consuming the bees themselves, which are highly important for the environment, biodiversity, and pollination [19]. The consumption of drone brood, however, has advantages for the hives and for the beekeepers, who usually discard this valuable resource [20]. The production of drone frames in the hive can have advantages for the welfare of the bees by helping control *Varroa* spp. mites, which are parasites that cause colony losses and diminish the productivity of the bees. The use of this technique constitutes a natural way of eliminating *Varroa*, avoiding the use of harmful chemical substances [20].

In the European market, this is highly regulated, and the types of insects that can be produced and commercialised as human food are established under the Regulation for Novel Foods by EFSA, the European Food Safety Authority. As of 2023, the Commission has authorised four insects: yellow mealworm (*Tenebrio molitor*), migratory locust (*Locusta migratoria*), cricket (*Acheta domesticus*), and lesser mealworm (*Alphitobius diaperinus*). Nevertheless, EFSA is carrying out safety assessments to possibly approve eight more insects [21]. To increase the number of accepted species in the European market, it is necessary to establish their nutritional relevance, as well as their safety, so they can be considered novel foods [21,22,23].

The possible addition of honeybee larvae/drones as novel foods has raised interest and motivated us to carry out this compilation of information on the nutritional value of larvae from *A. mellifera* species. Hence, the aim of this work was to review the research published in the scientific literature about the chemical composition and nutritional value of bee brood. The scientific databases were used to retrieve the works, and the information was analysed and categorised into different components with nutritional relevance.

## 2. Materials and Methods

This integrative literature review focuses on the chemical composition and nutritive and bioactive properties of the larvae and pupae of the *A. mellifera* L. The documents were searched in the scientific databases (ScienceDirect, Scopus, PubMed, BOn, and SciELO). Keywords used for the search included brood, larvae, pupae, *A. mellifera*, bee, drone, apilarnil, nutrition, fat, protein, amino acids, fatty acids, minerals, vitamins, bioactive compounds, phytochemicals, antioxidant, and phenolic compounds.

The inclusion/exclusion criteria consisted of the following:

- considering all documents that addressed the topics of this literature review;
- including documents published in the English language;
- excluding all documents whose authorship could not be confirmed;
- excluding works whose content was irrelevant to the present scope.

A total of 45 documents were included in this review, of which 40 were scientific manuscripts, either original research articles or reviews.

The documents included in the research were evaluated, and the most relevant information was extracted to build the present review. With respect to some of the topics addressed here, the number of research studies found is quite limited, which makes it difficult to find and report all the targeted information.

VOSviewer software, version 1.6.18, was used to analyse the bibliographic sources so as to recognise possible co-occurrences between the keywords used for indexing in each of the studies. This is a freely available program that can be downloaded and installed on any computer for free from the website https://www.vosviewer.com/ (accessed on 13 December 2024). In this work, the bibliographic sources included in the review were analysed for co-occurrence and links between the keywords and authors. This information is imported by the software based on the metadata of a list of bibliographic references.

**Figure 1:**
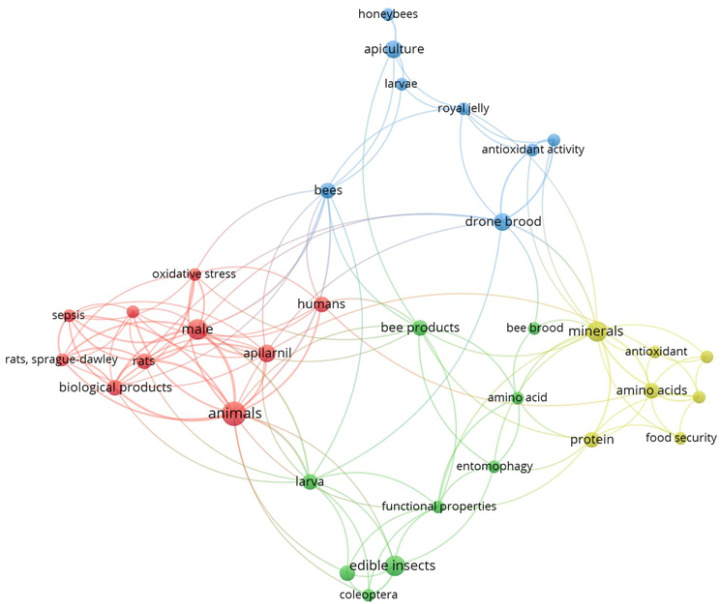
Analysis of co-occurrence links between keywords (Image created by Raquel Guiné using VOSviewer software, version 1.6.20).

**Figure 2:**
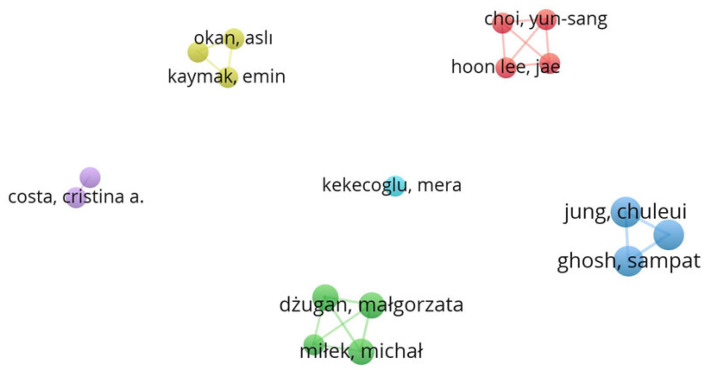
Analysis of co-occurrence links between authors (Image created by Raquel Guiné using VOSviewer software).

Figure 1 shows a schematic representation of the connections between keywords in the bibliographic sources included in this review. The dimensions of the circles and the sizes of the fonts for the corresponding labels directly relate to the relative frequency of occurrence for each of the keywords considered. The proximity of the circles represents the sources in which the keywords occur jointly. The absence of links indicates separate clusters where there is no connection between elements. The same applies to Figure 2, which deals with authors instead of keywords.

The analysis with the VOSviewer software produced a graph based on 180 keywords, of which 34 appeared at least twice. All of these were connected with other keywords, and therefore, they were included in the analysis. The representation is based on a cluster structure comprising four clusters (one of each colour—blue, red, green, yellow), with 128 links and a total link strength of 172. The keyword “animals” was highlighted as the most frequent keyword.

Figure 2 shows the schematic representation of the network between the authors of the works included in the review. Of the 196 authors, only 17 had a minimum of two publications. The graph in Figure 2 establishes six clusters, represented by different colours (yellow, red, lilac, cyan, blue, and green), with 19 links and a total link strength of 47, for the 17 items.

## 3. Results and Discussion

**Figure 3:**
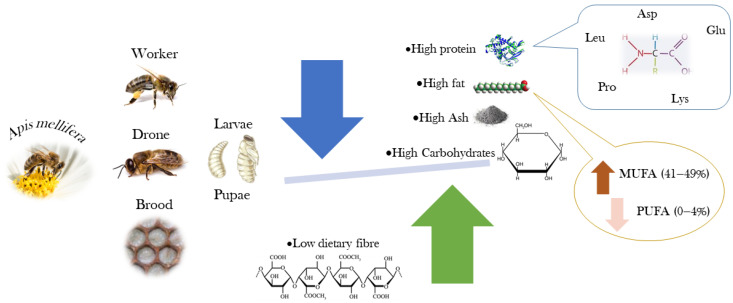
Nutritional relevance of *Apis mellifera* pupae and larvae (Image created by Raquel Guiné).

The most complete description of the chemical composition and nutritional value of the bee brood was presented by Finke [24], who reported the nutrient composition of the bee brood for potential use as human food. This work studied the macronutrient composition and energy value, complemented by the quantification of vitamins and minerals, amino acids, and fatty acids. Ghosh et al. [25] described the chemical composition of worker honey bees intended for human consumption in different developmental stages. The authors evaluated the proximate composition and energy value, the profiles of amino acids and fatty acids, and the mineral content. More recently, Haber et al. [3] evaluated the nutritional value (proximate composition) and the fatty acid profile of edible larvae and pupae of honey bees and related it to the bees’ diet. A review published by Ghosh et al. [26] presents data for the chemical composition of honey bee workers from different species and subspecies at different developmental stages. Figure 3 shows the nutritional relevance of *A. mellifera*, highlighting the presence of amino acids and monounsaturated fatty acids.

The results presented in Table 1 refer to the proximate composition of bee brood according to several studies, and they confirm that the brood constitutes a particularly rich source of protein, fat, and carbohydrates. However, the fibre content is low, and so is the level of ash [3,24,25].

In a recent review by Rutka et al. [17], some values for proximate composition of larvae and pupae are provided, based on the work by Ghosh et al. [25], as indicated in Table 1: 35.3% protein, 14.5% fat, 46.1% carbohydrates, and 4.1% ash for larvae, and 45.9% protein, 16.0% fat, 34.3% carbohydrates, and 3.8% ash for pupae (values expressed on a dry matter basis). These results indicate an increase in protein and fat from the larvae to the pupae stages of development, while the carbohydrate content decreases significantly. The results by Haber et al. [3] confirm the trend of previous results, with the pupae richer in protein when compared with the larvae. However, the values found in this study are higher for fat and protein when compared to those from Ghosh et al. [25]. The energy value varies between 1908 and 2019 kJ/100 g dry mass (Table 1), depending on the developmental stage and also varying between studies, possibly due to differences in the experimental methodologies applied.

**Table 1:** Centesimal composition and energy value of the bee brood of *A. mellifera* subspecies *ligustica* and others.

Macro Nutrients (g/100 g d.m.) ^1^	Larvae *A. mellifera ligustica* [25]	Larvae *A. mellifera* (Subspecies Not Indicated) [3]	Pupae *A. mellifera ligustica* [25]	Pupae *A. mellifera* (Subspecies Not Indicated) [3]	Brood *A. mellifera* (Subspecies Not Indicated) [24]	Pupae *A. mellifera* (Subspecies Not Indicated) [27]
Honey Bee Caste	Workers	Brood	Workers	Brood	Brood	Drone
Moisture (g/100 g)	74.4	—	79.3	—	76.8	—
Protein	35.3	19.0	45.9	24.6–26.6	40.5	48.5–51.8
Fat	14.5	28.1	16.0	19.1–21.1	20.3	25.8–26.2
Fibre (crude)	—	—	—	—	—	2.5–2.7
Fibre (acid detergent)	—	—	—	—	1.3	—
Fibre (neutral detergent)	—	—	—	—	0.9	—
Ash	4.1	2.8	3.8	3.5–3.2	3.4	4.0–4.2
Carbohydrates	46.1	50.1	34.3	80.8–51.1	34.5	15.4–15.9
Energy (kJ/100 g d.m.)	1908.0	—	1946.5	—	2019.0	—

^1^ All values are expressed in g/100 g dry matter (d.m.), except for moisture, which is expressed in g/100 g sample.

With respect to amino acid content, Table A1 in Appendix A presents detailed information on essential amino acids in bee brood, and Table A2 presents the non-essential amino acids and others that are conditionally essential amino acids for humans. A summary of the most relevant results is shown in Table 2 for easy understanding. The results show that leucine and lysine are the essential amino acids present in higher amounts, while glutamic acid, aspartic acid, and proline stand out as the most abundant non-essential amino acids [24]. According to Finke [24], the protein recovery from amino acids accounted for nearly 90% of the total nitrogen. In the review by Ghosh et al. [26], the data for amino acids of bee workers at different developmental stages (starting at prepupa and going through to late pupa) were presented, as resulting from previous studies by the same authors [28,29]. In all those studies, the results from Finke [24] were confirmed, with the most abundant essential amino acids being leucine, lysine, and aromatic amino acids, while the non-essential amino acid present in higher amounts is glutamic acid, a trend which is common to all *A. mellifera* subspecies.

Oliveira et al. [30] reported the amino acid content of two edible insects (*Tenebrio molitor* and *Gryllus assimilis*) and found that lysine was the limiting amino acid in both cases. The same authors reported a good digestibility of the proteins, and they also observed that the developmental stage was a factor affecting the amino acid profiles and functionality of proteins.

According to the World Health Organisation and the Food and Agriculture Organisation of the United Nations, protein intake has many health implications, for renal functioning, bone health, kidney stones, cardiovascular diseases, and cancer. Typical protein intake from food is up to 3 g per kg of body weight [31]. The same report of the WHO/FAO [31], stipulates the requirements for some indispensable amino acids, namely lysine (between 12 and 45 mg/kg body weight per day), leucine (between 14 and 40 mg/kg body weight per day), isoleucine and valine (both between 10 and 26 mg/kg body weight per day), threonine (between 7 and 20 mg/kg body weight per day), tryptophan (between 3.5 and 4 mg/kg body weight per day), and methionine and cysteine (both between 4 and 15 mg/kg body weight per day). For an adult, it is recommended to consume 59 mg of leucine per g of protein and 45 mg of lysine per g of protein. According to the values reported in Table 2, bee brood can supply an average of 87.5 mg of leucine per g of protein and 155 mg of lysine per g of protein, thus being higher than recommended.

**Table 2:** Most abundant amino acids present in the bee brood of *A. mellifera*, according to subspecies.

Subspecies of *A. mellifera* ^1^	Reference	Amino Acids (g/100 g Dry Matter)				
		Leucine ^2^	Lysine ^2^	Aspartic Acid ^3^	Glutamic Acid ^3^	Proline ^3^
*A. mellifera mellifera*	[26,28]	2.7–3.5	2.4–3.1	2.4–3.0	6.6–8.1	2.8–3.6
*A. mellifera ligustica*	[25,26]	2.5–5.5	1.9–4.4	2.5–3.5	5.0–12.2	3.0–4.6
*A. mellifera carnica*	[26,28]	2.6–3.6	2.3–3.2	2.4–2.8	6.3–7.4	2.4–3.7
*A. mellifera buckfast*	[26]	4.0–4.3	3.5–3.7	3.2	7.9–8.8	1.5–1.6

^1^ Different castes and different developmental stages. ^2^ Essential amino acids. ^3^ Non-essential amino acids.

Table 3 shows the amount of fatty acids according to the degree of saturation for *A. mellifera* castes and developmental stages. The complete values for the subspecies of *A. mellifera* in different developmental stages are given in detail in Table A3, Table A4 and Table A5, respectively, for saturated, monounsaturated, and polyunsaturated fatty acids.

Higher values of total fatty acids are found in larvae and pupae of *A. mellifera mellifera*, more than twice as much as in prepupae or early pupae of the same subspecies, or even when compared with other subspecies of *A. mellifera*. Total fatty acids are lowest for the late pupa of *A. mellifera carnica.* Monounsaturated fatty acids are present in the highest amounts also in larvae and pupae of *A. mellifera mellifera*. As for the polyunsaturated fatty acids, they are also highest for larvae and pupae of subspecies *mellifera,* while being absent in larvae and pupae of subspecies *ligustica* and prepupae of subspecies *buckfast.* Bee brood presents a low ratio of polyunsaturated fatty acids (lower than 4%), which is not aligned with the relative abundance of these important fatty acids in other edible insects, such as *Tenebrio molitor* (up to 77% PUFAs).

The two primary fatty acids in bee brood are oleic acid (monounsaturated) and palmitic acid (saturated). Most of the fat is saturated (51.8%) and monounsaturated (46.2%) fatty acids, with the polyunsaturated fatty acids present in vestigial amounts (2.0%) [24]. Ghosh et al. [25] confirmed the observations by Finke [24] that most fat consists of saturated and monounsaturated fatty acids. According to more recent studies [26,28] for different developmental stages of *A. mellifera* subspecies, the polyunsaturated fatty acids (C18:2 and C18:3) also represent a minor portion of the fat, regardless of the developmental stage, with oleic acid (C18:1) and palmitic acid (C16:0) showing the highest amounts [29,32]. It was observed that most fatty acids are saturated (corresponding to more than 50%), followed by monounsaturated fatty acids, with the polyunsaturated fatty acids present only in minor fractions (about 1%) [26]. This trend was similar across all subspecies. The most abundant fatty acids were identified as the palmitic acid (C16:0), oleic acid (C18:1), and stearic acid (C18:0).

**Table 3:** Total amount of fatty acids and amounts of saturated, monounsaturated, and polyunsaturated fatty acids of the bee brood from different *A. mellifera* subspecies.

Species/Subspecies and Developmental Stage	Honey Bee Caste	Ref.	Fatty Acids (mg/100 g Dry Matter) (%)			
			SFAs ^1^	MUFAs ^2^	PUFAs ^3^	Total FAs ^4^
*A. mellifera mellifera* Larvae	Brood	[3]	14,021.9 (50%)	12,897.9 (46%)	1152.1 (4%)	28,071.9 (100%)
*A. mellifera mellifera* Pupae ^5^	Brood	[3]	9359.0–10,022.5 (49–48%)	8900.6–10,275.7 (47–48%)	780.7–821.3 (4–4%)	19,080.9–21,078.9 (100%)
*A. mellifera mellifera* Prepupae	Drones	[26,28]	6483.5 (58%)	4521.6 (41%)	149.4 (1%)	11,154.6 (100%)
*A. mellifera mellifera* Early (white-eyed) pupae	Drones	[26,28]	6450.3 (57%)	4654.7 (41%)	197.7 (2%)	11,302.7 (100%)
*A. mellifera mellifera* Late (dark-eyed) pupae	Drones	[26,28]	5396.9 (55%)	4264.2 (43%)	223.8 (2%)	9884.9 (100%)
*A. mellifera ligustica* Larvae	Drones	[25]	2560.6 (52%)	2381.2 (48%)	0.0 (0%)	4941.8 (100%)
*A. mellifera ligustica* Early (white-eyed) pupae	Drones	[26]	6414.1 (56%)	4965.9 (43%)	99.2 (1%)	11,479.2 (100%)
*A. mellifera ligustica* Late (dark-eyed) pupae	Drones	[26]	5341.1 (54%)	4470.7 (45%)	131.2 (1%)	9943.0 (100%)
*A. mellifera ligustica* Pupae	Workers	[25]	2821.1 (51%)	2663.2 (49%)	0.0 (0%)	5484.3 (100%)
*A. mellifera carnica* Prepupae	Drones	[26,28]	6453.7 (56%)	4766.9 (42%)	228.6 (2%)	11,449.2 (100%)
*A. mellifera carnica* Early (white-eyed) pupae	Drones	[26,28]	6475.8 (56%)	4831.7 (42%)	239.0 (2%)	11,546.5 (100%)
*A. mellifera carnica* Late (dark-eyed) pupae	Drones	[26,28]	4885.4 (51%)	4373.3 (46%)	242.8 (3%)	9501.5 (100%)
*A. mellifera buckfast* Prepupae	Drones	[26]	6305.7 (57%)	4776.6 (43%)	0.0 (0%)	11,082.3 (100%)
*A. mellifera buckfast* Late (dark-eyed) pupae	Drones	[26]	6634.6 (56%)	5156.4 (43%)	67.0 (1%)	11,858.9 (100%)

^1^ SFA = saturated fatty acid; ^2^ MUFA = monounsaturated fatty acid; ^3^ PUFA = polyunsaturated fatty acid; ^4^ FA = fatty acid. ^5^ Range of values corresponding to pupae fed with different diets.

The results in Table 3 report the relative percentages of the different fractions of fatty acids, with saturated fatty acids representing between 48% and 58%, monounsaturated fatty acids representing between 41% and 49%, and polyunsaturated fatty acids representing between 0% and 4% of total fatty acids. Hence, saturated and monounsaturated fatty acids are quite comparable, each constituting about half of the total fatty acids, while polyunsaturated fatty acids represent a very low fraction. Raksakantong et al. [33] analysed the fatty acid profiles in eight edible insects and reported percentages of saturated fatty acids in the range of 29–56%, monounsaturated fatty acids in the range of 1–7%, and polyunsaturated fatty acids in the range of 43–69%. These results represent a much higher fraction of polyunsaturated fatty acids than those found in the bee brood. A recent meta-analysis by Palupi et al. [34] reported ratios between polyunsaturated fatty acids and monounsaturated fatty acids varying between a minimum of 0.01 for African palm weevil and a maximum of 1.34 for house cricket.

**Figure 4:**
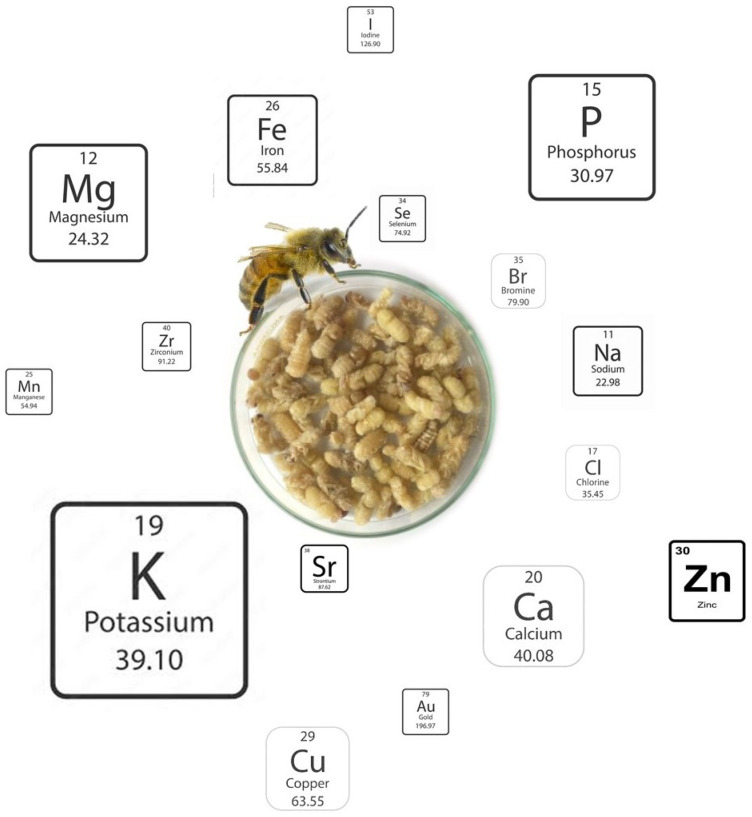
Relative abundance (according to the proportional size) of minerals in bee brood (Image created by Raquel Guiné).

Table A6 shows the detailed mineral content of the brood of *A. mellifera* subspecies, again considering different developmental stages based on the values reported in several studies [24,25,28,29]. The results were obtained from the different studies, expressed in variable units, and converted to dry matter for ease of analysis. Some macro minerals are present in all subspecies of *A. mellifera* in relevant quantities, like potassium, phosphorus, magnesium, and calcium (Figure 4). These minerals are of dietary importance for several body functions in humans: Potassium contributes to maintaining healthy nerve and muscle function, regulating heartbeat, and controlling fluid balance within cells [35]. Phosphorus participates in bone mineralisation, energy metabolism, and cell signalling and contributes to brain health [36]. Magnesium acts as a cofactor for over 300 enzymatic reactions, including those involved in protein synthesis, cellular energy production, and DNA/RNA synthesis; it also plays a crucial role in nerve and muscle function, including nerve transmission, muscular contraction, and cardiac excitability [37]. Calcium has a vital role in numerous bodily functions, being best known for its role in maintaining strong bones and teeth; beyond skeletal health, calcium is crucial for muscle contraction, nerve transmission, blood clotting, and cell signalling, and is associated with the prevention of ischemic stroke and heart failure, cancers, type 2 diabetes, and Parkinson’s disease [38,39]. On the other hand, sodium and calcium are much more abundant in the larvae of *A. mellifera ligustica* than in all other broods analysed. With respect to trace minerals, Finke [12] reported that the brood possesses relevant quantities of iron, copper, selenium, and zinc, as well as low amounts of iodine and manganese. When comparing the studies that report the mineral content of bee brood, substantial differences were encountered, although the relative proportion of the different elements was generally similar. Finally, a trend of increasing mineral content is observed along the development of the brood, from the larva to the late pupa stages.

The most relevant heavy metals found in bee brood were copper and zinc, according to the results in Table A7. Only one work reported the presence of bismuth, lead, molybdenum, and silver. In the study by Borkovcová et al. [40], who analysed drone bees collected from an industrial area (Opava, in the Czech Republic), the content of lead was considered high (0.21 mg/100 g), and the authors considered that this level of heavy metals could be a consequence of the industrial activity. Edible insects, while a promising alternative protein source, can accumulate heavy metals, posing potential health risks. The levels of heavy metals like arsenic, cadmium, mercury, and lead can vary depending on the insect species, their diet, developmental stage, and environmental conditions. While some studies show heavy metal concentrations in edible insects remain below regulatory limits, others highlight the potential for increased heavy metal intake through insect consumption, especially when insects are raised on contaminated substrates [41,42]. Schrögel and Wätjen [42] reported some cases of accumulation of heavy metals in edible insects, including the accumulation of cadmium in black soldier fly and arsenic in yellow mealworm.

Fialho et al. [43] evaluated the nutritional composition of larvae of mealworm (*Tenebrio molitor*) and crickets (*Gryllus assimilis*) and reported that the most abundant macro minerals were potassium and phosphorus, just like in the case of bee brood. Also, they reported that mealworms and crickets were low in sodium, calcium, and magnesium and contained some trace minerals like iron, manganese, zinc, and copper [43]. Oliveira et al. [30] analysed the mineral content in two edible insects (*Tenebrio molitor* and *Gryllus assimilis*) and reported the presence of magnesium, phosphorus, and potassium in higher contents, but also copper, calcium, iron, zinc, manganese, and sodium. These results highlight the similarity in nutritional value in terms of minerals with dietary relevance between bee broods and other edible insects.

The results in Table 4 present the vitamin content of honey bee pupae in different developmental stages, the most complete analysis being by Finke [24]. The results for vitamin A show nearly undetectable levels, according to Finke [24] and Hu and Li [44]. Other fat-soluble vitamins (D and E) are also present in small amounts. On the other hand, bee brood is rich in vitamin C, with vitamins of the B group also present in appreciable amounts. Choline, a nutrient similar to the vitamins in the B group, is also present. Even though the details are not the same in the different studies [24,44,45], with some studies more complete than others, some results are worth highlighting when it comes to the development of the pupae, such as the increase in vitamin levels along the development of the worker larvae from day 9 to day 19 [44].

Oliveira et al. [30] evaluated the nutritional value of two edible insects (*Tenebrio molitor* and *Gryllus assimilis*) and found that they are good sources of niacin and vitamin C, as is the case with bee brood. However, they reported higher amounts of vitamin E in the yellow mealworm beetle and cricket when compared with bee brood, but they did not detect riboflavin, which is present in bee brood.

**Table 4:** Vitamin content of brood *A. mellifera* subspecies *ligustica* and others.

Vitamins (μg/100 g) ^1^	Worker Larvae ^2^ *A. mellifera ligustica* [44]	Drone Pupae (Subspecies Not Indicated) [45]	Brood (Subspecies Not Indicated) [24]
Honey Bee Caste	Workers	Drones	Brood
Vitamin A	1.32–7.41	n.d.	<100 ^3^
Beta-carotene (provitamin A)	n.a.	n.a.	<20
Vitamin B_1_ (thiamine)	0.94–3.27	1550	410
Vitamin B_2_ (riboflavin)	0–251	2930	910
Vitamin B_3_ (niacin)	n.a.	n.a.	3670
Vitamin B_5_ (pantothenic acid)	n.a.	n.a.	1190
Vitamin B_6_ (pyridoxine)	n.a.	n.a.	120
Vitamin B_7_ (biotin)	n.a.	n.a.	0.023
Vitamin B_9_ (folic acid)	n.a.	n.a.	<6
Vitamin B_12_	n.a.	n.a.	<0.12
Vitamin C	4020–4350	n.a.	3800
Vitamin D	390–410	n.d.	<25.1 ^3^
Vitamin E	0.87–1.10	n.a.	<0.5 ^3^
Choline	n.a.	n.a.	168,400

n.a. = not available; n.d. = not detected. ^1^ Except if other units are presented. ^2^ Range of values for different developmental stages. ^3^ IU/100 g.

Sidor et al. [13] evaluated the antioxidant activity of drone brood obtained from three apiaries (Poland), depending on the stage of larval development (7th, 11th, and 14th days). As observed in Table 5, the antioxidant potential of drone brood homogenate depends on the stage of larval development, with an increase between 7 and 11 days, followed by a reduction at later stages (14 days). However, Ghosh et al. [29] state that adult drones exhibit stronger antioxidant activity due to their higher phenolic content than larvae. In fact, Abedelmaksoud et al. [46] found that adult drones contain higher levels of total phenolics (9.243 mg GAE/g), antioxidant activity (3.206 mg TE/g), and total flavonoids (2.215 mg CE/g) compared to drone pupae, which presented 5.708 mg GAE/g, 3.034 mg TE/g, and 0.524 mg CE/g, respectively.

Furthermore, flavonoid content varied across developmental stages, with concentrations of 5.8 mg/g in larvae, 4.7 mg/g in pupae, and 4.5 mg/g in adult drones. Drone adults also have a higher total phenolic content (9.243 mg GAE/g) than drone pupa (5.708 mg GAE/g).

A comparison of extraction solvents reveals that the use of 70% ethanol resulted in a reduction of 48% in Fe (III) ion-reducing capacity across all developmental stages, as well as a reduction of phenolic and flavonoid compounds content in frozen drone brood [47]. These results are consistent with those of Sarıguzel et al. [48], who reported that the phenolic content of water-extracted drone larvae (7 days old) was 261.2 ± 16.6 mg GAE/100 g, whereas the ethanol (70%) extract contained only 92.4 ± 12.7 mg GAE/100 g. Similarly, for antioxidant activity, the anti-DPPH activity of the water extract of drone larvae was 16.2 ± 1.2%, while that of the ethanol extract was 4.8 ± 0.6%.

Sidor et al. [13] also reported that the use of freeze drying to preserve drone brood homogenate (apilarnil) did not show significant differences in antioxidant activity (a decrease of 6–10%) or total phenolic compounds (a decrease of 7–11%) when compared to frozen brood on a dry mass basis. Moreover, Sidor et al. [13] consider that using drone brood up to 10 days of age offers technological advantages, primarily due to the ease of removing larvae from uncapped patch cells.

**Table 5:** Total phenolic and flavonoid content and antioxidant activity of frozen drone brood homogenates extracted with water and 70% ethanol [13].

	Extract Solvent	Drone Brood (*A. mellifera carnica*) 7–11–14-Day-Old Larvae
Total phenolic content (mg GAE/100 g)	water	251.3–289.4–245.2
	ethanol (70%)	89.1–81.8–87.0
Total flavonoid content (mg GAE/100 g)	water	8.7–10.9–6.7
	ethanol (70%)	0.7–1.0–1.0
FRAP (mmol/100 g)	water	1.0–1.1–0.9
	ethanol (70%)	0.5–0.5–0.5
Antioxidant activity by ABTS method (%)	water	12.7–6.3- 11.6
	ethanol (70%)	20.4–21.0–20.7
Antioxidant activity by DPPH method (%)	water	14.2–20.1–14.7
	ethanol (70%)	4.3–4.0–3.0

FRAP = ferric reducing antioxidant power; ABTS = 2,2′-azino-bis (3ethylbenzothiazoline-6-sulfonic acid; DPPH-2,2-diphenyl-1-picrylhydrazyl).

Table 6 shows total phenolic and flavonoid contents and antioxidant activity of the brood drone of *A. mellifera* subspecies, based on the values reported in several studies [49,50,51]. The differences observed in the values depend on many factors, namely distinct environments and ecological characteristics, bee species, the geography where the bee lives, and the different food plants used by the bees.

**Table 6:** Total phenolic and flavonoid content and antioxidant activity of drone larvae.

	Drone Brood (4–9 Days After Hatching) [51]	Carniolan Hybrid Honeybees (*A. mellifera*) (7 Days Old) [50]	Drone Larvea Apilarnil (*A. mellifera anatoliaca)* [49]
Country of origin	Turkey	Egypt	Turkey
Total phenolic content	13.18 ± 0.19 *	n.a	14.35 ± 3.2 **
Total flavonoid (mg QE/100 g)	n.a.	13.16 ± 0.94	47.5 ± 3.62
FRAP activity (μmol Trolox/g)	27.17 ± 0.28	n.a.	
ABTS (mmol Trolox/100 g)	n.a.	n.a.	0.59 ± 12.73
DPPH radical scavenging activity (IC50: mg/mL)	0.65 ± 0.01	0.180 ±0.0025	n.a.

* mg GAE/g; ** mg GAE/100 g extract.

Table 7 presents the phytochemical compounds identified and quantified in apilarnil extracts using LC/MS/MS [52]. The main phytochemical compound of apilarnil is quinic acid (1091.045 mg/g dry extract), followed by fumaric acid (48.714 mg/g dry extract), aconitic acid (47.218 mg/g dry extract), kaempferol (39.946 mg/g dry extract), quercetin (27.508 mg/g dry extract), and several other bioactive compounds that are also present in lower concentrations.

The most abundant compounds found in apilarnil—such as quinic acid, fumaric acid, and aconitic acid—are commonly present in food and cosmetic products and are recognised for their significant biological activity, namely their antimicrobial and antioxidant properties [14]. In a study by Inci et al. [52], apilarnil demonstrated superior DPPH^•^ radical scavenging activity (IC_50_: 40.764 μg/mL) compared to ascorbic acid (IC_50_: 49.500 μg/mL). Furthermore, apilarnil effectively inhibited the activity of several key enzymes, including human carbonic anhydrase I (hCA I, IC_50_: 14.2 μg/mL), hCA II (IC_50_: 11.5 μg/mL), acetylcholinesterase (AChE, IC_50_: 21.1 μg/mL), and butyrylcholinesterase (BChE, IC_50_: 16.1 μg/mL), indicating its potential for therapeutic applications.

Given these characteristics, drone brood represents a promising candidate for use as a food product or functional food ingredient. An example is the innovative dietary supplement proposed by Dżugan et al. [53], which consists of freeze-dried drone brood enriched with organic calcium derived from eggshells. The synergistic interaction between the bioactive components of drone brood and the calcium content of eggshells appears to be an effective approach to addressing hormonal and calcium deficiencies, particularly in the context of osteoporosis treatment. Additional applications include the incorporation of drone brood homogenate in functional beverages and its use in the prophylaxis and management of fatigue-related conditions. Furthermore, formulations have been proposed in which drone brood is used as a food supplement recipe designed to increase muscle mass in athletes [12]. The richness of bioactive compounds found in apilarnil suggests it could be a valuable addition to functional foods, offering potential health and wellness benefits.

The results in this review highlight the benefits of consuming bee brood due to their nutritive and bioactive properties, such as macro and micro nutrients (fat and fatty acids, protein and amino acids, fibre, vitamins, and minerals) and also bioactive compounds (like phenolic compounds) and antioxidant activity. All these components present nutritive and health benefits, which make bee brood a valuable dietary element. However, further research is necessary to understand the properties of these ingredients fully, confirm their effectiveness, determine the dose-related toxicity during in vivo studies, and ensure their safety. One other issue that needs to be addressed is microbiological safety, which is essential to ensure that the bee brood is safe for human consumption. Possible microbial risks like bacterial contamination (for example, with *Salmonella*, *Listeria*, or *E. coli*) or fungal growth need to be assessed. If bee brood is contaminated with *C. botulinum* and is not properly processed, this may cause severe toxicological problems. It is true that processing methods such as boiling and drying can inactivate most bacterial pathogens, but spore-forming microorganisms like *Clostridium* and *Bacillus* are frequently resistant to mild thermal treatments [54]. The review by Garofalo et al. [55] presents the scientific evidence related to the microbiota of edible insects, highlighting the possible risks for human health. They alert that raw edible insects usually contain high loads of mesophilic aerobes, bacterial endospores or spore-forming bacteria, Enterobacteriaceae, lactic acid bacteria, psychrotrophic aerobes, and fungi. Also, Cruz-Garcia et al. [54] focus on the biosafety challenges of edible insects consumed in Mexico. Edible insects can be consumed at various life stages (egg, larva, pupa, adult), with the larvae and pupae being the most commonly consumed forms. Hence, possible contamination will affect humans upon consumption. Another review by Murefu et al. [56] reports on the safety of edible insects harvested in the wild versus those that are farmed under more controlled conditions, highlighting the role of processing methods like boiling, frying, and roasting in significantly increasing the safety of the edible insects. For these reasons, it is relevant to investigate the possible microbiological contamination of bee brood to ensure safe consumption.

One other problem that can be associated with the consumption of edible insects is the presence of substances that are allergens and can cause problems to some consumers. Allergens like tropomyosin and arginine kinase can be present in edible insects. A review by Ribeiro et al. [57] discusses the allergic risks of consuming edible insects, referring to case reports of anaphylaxis and allergy. Processing can affect the solubility and the immunoreactivity of the allergens present in insects, with variable effectiveness depending on species and type of proteins involved. In some cases, chemical or enzymatic hydrolysis can eliminate immunoreactivity [58]. Bee pupae and bee larvae have been reported to pose a risk of anaphylactic shock in China, with one reported case for each [59].

**Figure 5:**
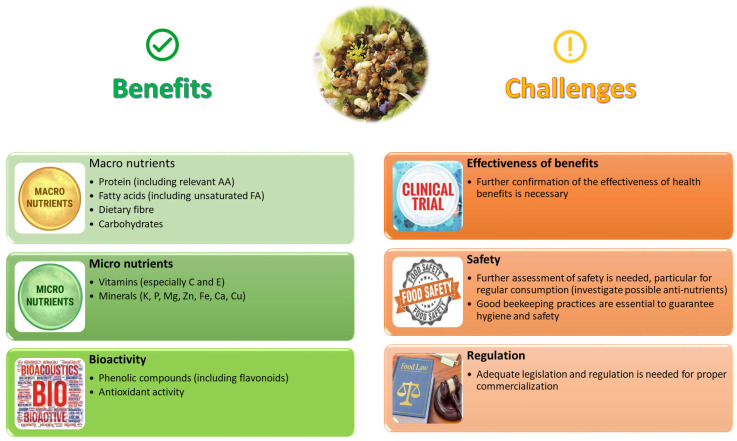
Pros and cons of using bee brood as a food for humans (Image created by Raquel Guiné).

The marketing of drone brood as a food or food supplement requires appropriate legislation from governmental authorities and approval for its commercial use. It is important to note that a standard dossier of beekeeping practices is essential to ensure the hygiene of the honeybee drone production and to address potential safety issues. Once these obstacles are overcome, the use of drones as food or feed can be a win–win solution for beekeepers as well as consumers. Figure 5 summarises some pros and cons of bee brood as a human food based on its phytochemical and nutritional composition, while focusing on some understudied topics and challenges for the future, where further research is still necessary.

## 4. Conclusions

The results of this review highlight that larvae, pupae, and broods of *A. mellifera* are rich in protein, fat, and carbohydrates and have a high energy value per 100 g of edible portion. Concerning the amino acids of the proteins present in bee brood, the most abundant essential amino acids are leucine and lysine, while the most abundant non-essential amino acids are aspartic acid, glutamic acid, and proline. Regarding the quality of the fat present in bee brood, the relative percentages of saturated fatty acids and monounsaturated fatty acids are approximately equal, each accounting for around 50%, while the percentage of polyunsaturated fatty acids is very low, below 4%. The dietary minerals present in higher quantities are potassium, phosphorus, magnesium, calcium, iron, copper, zinc, and sodium. In what concerns the vitamins, bee brood is rich in choline, vitamin C, and some vitamins of the B complex like niacin (B3), pantothenic acid (B5), and riboflavin (B2).

These results indicate a potential for the utilisation of bee brood in human diets due to the richness of some important nutrients for proper body functioning. Additionally, the richness in phytochemical substances with biological activity turns this resource into a valuable ally in promoting human health. However, some aspects still need to be further studied since the number of studies reporting results is still scarce, for example, the evaluation of vitamins or the evaluation of bioactive compounds. On the other hand, the possible presence of anti-nutrients, which are sometimes present in some edible insects, also needs to be studied in the future. The safety aspects linked with bee brood consumption are also a topic that should be addressed in future studies, namely, possible adverse effects caused by their consumption, including allergies, or hazards that could be linked to their utilisation as food, including the presence of heavy metals or microbiological safety. Also, additional research would be important to confirm the usefulness of brood as a therapeutic agent to promote human health.

## Figures and Tables

**Table 7:** Phytochemical compounds identified and quantified in apilarnil extracts [52].

Phytochemical Compounds	Amount (mg/g Dry Extract)
Quinic acid	1091.045
Fumaric acid	48.714
Aconitic acid	47.218
Kaempferol	39.946
Quercetin	27.508
4-OH-Benzoic acid	16.307
Protocatechuic acid	0.769
Chlorogenic acid	0.905
p-Coumaric acid	9.023
Caffeic acid	2.011
Quercitrin	4.553
Luteolin	2.747
Hesperetin	2.671
Nicotiflorin	2.461
Caffeic acid	2.011
Vanillin	1.37
Acacetin	1.25
Amentoflavone	0.981
Chlorogenic acid	0.905
Chrysin	0.884
Astragalin	0.866
Protocatechuic acid	0.769
Isoquercitrin	0.757
Naringenin	0.551
Cosmosiin	0.249
Apigenin	0.153

## Data Availability

No new data were created or analyzed in this study. Data sharing is not applicable to this article.

## Appendix A. Complete Tables for Amino Acids, Fatty Acids, and Minerals

Table A1 and Table A2 present the amounts of essential and non-essential amino acids, respectively, in several subspecies of bee brood.

Table A3, Table A4 and Table A5 refer to the contents of saturated, monounsaturated and polyunsaturated fatty acids, respectively, in different subspecies of bee brood.

Table A6 and Table A7 show, respectively, the amounts of the different minerals and heavy metals in different subspecies of bee brood.

**Table A1:** Essential amino acids in the bee brood of A. *mellifera* subspecies *mellifera, ligustica, carnica, buckfast*, and others.

Species/Subspecies and Developmental Stage, When Available	Honey Bee Caste	Reference	Essential Amino Acids (g/100 g Dry Matter)								
			Histidine	Isoleucine	Leucine	Lysine	Methionine	Phenylalanine	Threonine	Tryptophan	Valine
Prepupae *Apis mellifera mellifera*	Drones	[26,28]	0.8	1.6	2.7	2.4	0.5	1.5	1.4	n.a.	1.9
Early (white-eyed) pupae *A. mellifera mellifera*	Drones	[26,28]	0.9	1.9	3.1	2.8	0.8	1.6	1.5	1.5	2.2
Late (dark-eyed) pupae *A. mellifera mellifera*	Drones	[26,28]	1.1	2.2	3.5	3.1	0.8	1.8	1.7	1.7	2.4
Larvae *A. mellifera ligustica*	Workers	[25]	0.7	1.6	2.5	1.9	n.a.	0.2	1.6	n.d.	1.7
Prepupae *A. mellifera ligustica*	Drones	[26]	0.9	2.1	3.5	3.0	n.a.	n.a.	1.9	n.a.	2.6
Early (white-eyed) pupae *A. mellifera ligustica*	Drones	[26]	1.1	2.4	4.1	3.5	n.a.	n.a.	1.9	n.a.	3.0
Late (dark-eyed) pupae *A. mellifera ligustica*	Drones	[26]	1.4	3.2	5.5	4.4	n.a.	n.a.	3.2	n.a.	4.1
Pupae *A. mellifera ligustica*	Workers	[25]	1.1	2.3	3.2	3.0	n.a.	0.2	1.9	n.d.	2.4
Prepupae *A. mellifera carnica*	Drones	[26,28]	0.8	1.6	2.6	2.3	0.4	1.4	1.3	n.a.	1.8
Early (white-eyed) pupae *A. mellifera carnica*	Drones	[26,28]	0.9	1.9	3.2	2.8	0.2	1.7	1.6	n.a.	2.2
Late (dark-eyed) pupae *A. mellifera carnica*	Drones	[26,28]	1.1	2.2	3.6	3.2	0.4	1.8	1.7	n.a.	2.5
Prepupae *A. mellifera buckfast*	Workers	[26]	1.2	2.4	4.0	3.5	n.a.	n.a.	1.9	n.a.	2.9
Late (dark-eyed) pupae *A. mellifera buckfast*	Drones	[26]	1.3	2.6	4.3	3.7	n.a.	n.a.	1.6	n.a.	3.0
Brood (subspecies not indicated)	Drones	[24]	0.9	1.9	2.8	2.5	0.9	1.4	1.3	0.4	2.1

n.a. = not available; n.d. = not detected.

**Table A2:** Non-essential amino acids and others in the bee brood of *A. mellifera* subspecies *mellifera, ligustica, carnica, buckfast*, and others.

Species/Subspecies and Developmental Stage, When Available	Honey Bee Caste	Reference	Non-Essential Amino Acids (g/100 g Dry Matter)								Others (g/100 g Dry Matter)				
			Alanine	Arginine	Aspartic Acid	Cysteine	Glutamic Acid	Glycine	Proline	Serine	Tyrosine ^2^	Taurine ^3^	Sulphur-containing AA ^4^	Aromatic AA ^5^	Ammonia ^6^
Prepupae *A. mellifera mellifera*	Drones	[26,28]	1.5	1.7	2.4	0.5	6.6	1.6	2.8	1.4	1.5	n.a.	1.8	n.a.	n.a.
Early (white-eyed) pupae *A. mellifera mellifera*	Drones	[26,28]	2.0	2.0	2.8	0.6	7.6	1.9	3.3	1.7	1.8	n.a.	1.4	3.4	n.a.
Late (dark-eyed) pupae *A. mellifera mellifera*	Drones	[26,28]	2.5	2.3	3.0	1.0	8.1	2.4	3.6	2.0	2.1	n.a.	1.8	3.9	n.a.
Larvae *A. mellifera ligustica*	Workers	[25]	1.6	1.6	2.6	0.3	5.0	1.4	n.a.	1.4	1.5	n.a.	1.0	n.a.	n.a.
Prepupae *A. mellifera ligustica*	Drones	[26]	2.6	2.2	2.5	n.a.	10.0	2.1	3.0	1.8	n.a.	n.a.	0.4	4.0	n.a.
Early (white-eyed) pupae *A. mellifera ligustica*	Drones	[26]	3.4	2.6	2.7	n.a.	10.6	2.8	3.6	2.1	n.a.	n.a.	0.7	4.8	n.a.
Late (dark-eyed) pupae *A. mellifera ligustica*	Drones	[26]	5.8	3.4	3.2	n.a.	12.2	4.6	4.6	3.2	n.a.	n.a.	2.3	5.3	n.a.
Pupae *A. mellifera ligustica*	Workers	[25]	2.9	2.3	3.5	0.4	8.4	2.5	n.a.	2.0	2.0	n.a.	n.a.	n.a.	n.a.
Prepupae *A. mellifera carnica*	Drones	[26,28]	1.5	1.7	2.4	0.2	6.3	1.5	2.4	1.4	1.6	n.a.	1.4	3.0	n.a.
Early (white-eyed) pupae *A. mellifera carnica*	Drones	[26,28]	2.0	2.1	2.8	0.1	7.7	1.9	3.0	1.6	1.7	n.a.	0.3	3.4	n.a.
Late (dark-eyed) pupae *A. mellifera carnica*	Drones	[26,28]	2.9	2.3	2.8	0.7	7.4	2.6	3.7	1.9	2.0	n.a.	1.1	3.8	n.a.
Prepupae *A. mellifera buckfast*	Workers	[26]	2.4	2.2	3.2	n.a.	7.9	2.3	1.6	2.0	n.a.	n.a.	1.4	4.6	n.a.
Late (dark-eyed) pupae *A. mellifera buckfast*	Drones	[26]	2.9	2.5	3.2	n.a.	8.8	2.7	1.5	2.4	n.a.	n.a.	1.5	4.9	n.a.
Brood (subspecies not indicated)	Drones	[24]	1.9	1.7	3.3	0.9 ^1^	5.6	1.8	2.5	1.4	1.8	0.1	n.a.	n.a.	0.8

n.a. = not available. ^1^ In the work by Finke [24] was reported the value for cysteine, which is the oxidised dimer form of the non-essential amino acid cysteine. ^2^ Tyrosine is a conditional essential amino acid for humans [25]. ^3^ Taurine is a conditional amino acid resulting from cysteine metabolism [60]. ^4^ Methionine, cysteine, homocysteine, and taurine are the four common sulphur-containing amino acids. However, only methionine and cysteine are incorporated into proteins [61]. ^5^ Aromatic amino acids include phenylalanine, tryptophan, and tyrosine [62]. ^6^ Ammonia results from glutamine and alanine metabolism [63].

**Table A3:** Saturated fatty acids of *A. mellifera* subspecies *mellifera, ligustica, carnica*, and *buckfast*.

Species/Subspecies and Developmental Stage, When Available	Honey Bee Caste	Reference	Saturated Fatty Acids (g/100 g Dry Matter)								
			Capric Acid (C10:0)	Lauric Acid (C12:0)	Myristic Acid (C14:0)	Palmitic Acid (C16:0)	Margaric Acid (C17:0) ^2^	Stearic Acid (C18:0)	Arachidic Acid (C20:0)	Behenic Acid (C22:0)	Lignoceric Acid (C24:0)
Larvae *A. mellifera*	Brood	[3]	n.a.	n.a.	843.0	9694.5	n.a.	2922.4	281.0	281.0	n.a.
Pupae ^1^ *A. mellifera*	Brood	[3]	n.a.	n.a.	506.4 to 515.7	5959.2 to 6055.7	n.a.	2158.3 to 2637.5	343.8 to 379.8	382.0 to 443.1	n.a.
Prepupae *A. mellifera mellifera*	Drones	[26,28]	n.d.	20.9	341.7	4847.7	4.3	1207.0	45.1	16.9	n.a.
Early (white-eyed) pupae *A. mellifera mellifera*	Drones	[26,28]	n.d.	24.9	354.0	4726.6	4.7	1260.0	58.9	21.2	n.d.
Late (dark-eyed) pupae *A. mellifera mellifera*	Drones	[26,28]	1.8	26.0	284.1	3803.9	4.5	1181.4	67.7	27.6	n.a.
Larvae *A. mellifera ligustica*	Drones	[25]	n.d.	15.1	116.6	1844.0	n.a.	584.9	n.a.	n.a.	n.a.
Early (white-eyed) pupae *A. mellifera ligustica*	Drones	[26]	n.d.	32.5	333.1	4517.5	n.d.	1356.9	120.6	14.4	39.2
Late (dark-eyed) pupae *A. mellifera ligustica*	Drones	[26]	n.d.	33.4	258.1	3570.8	n.d.	1267.0	145.8	23.3	42.6
Pupae *A. mellifera ligustica*	Workers	[25]	n.d.	24.6	157.5	1942.2	n.a.	696.8	n.a.	n.a.	n.a.
Prepupae *A. mellifera carnica*	Drones	[26,28]	2.0	28.2	379.3	4699.2	4.2	1277.9	46.8	16.0	n.a.
Early (white-eyed) pupae *A. mellifera carnica*	Drones	[26,28]	1.9	29.8	355.0	4640.6	4.1	1362.7	60.9	20.9	n.d.
Late (dark-eyed) pupae *A. mellifera carnica*	Drones	[26,28]	2.0	27.6	234.7	3307.0	4.1	1207.3	72.4	30.3	n.a.
Prepupae *A. mellifera buckfast*	Drones	[26]	n.d.	26.0	359.5	4810.0	n.d.	1110.3	n.d.	n.d.	n.d.
Late (dark-eyed) pupae *A. mellifera buckfast*	Drones	[26]	n.d.	31.4	365.5	4879.1	n.d.	1302.5	56.2	n.d.	n.d.

n.a. = not available; n.d. = not detected. ^1^ Range of values corresponding to pupae fed with different diets. ^2^ Margaric acid or Heptadecanoic acid.

**Table A4:** Monounsaturated fatty acids of *A. mellifera* subspecies *mellifera, ligustica, carnica*, and *buckfast*.

Species/Subspecies and Developmental Stage, When Available	Honey Bee Caste	Reference	Monounsaturated Fatty Acids (g/100 g Dry Matter)					
			Myristoleic Acid (C14:1)	Hexadecenoic Acid (C16:1t) ^2^	Palmitoleic Acid (C16:1) ^2^	Elaidic Acid (C18:1t) ^3^	Oleic Acid (C18:1) ^3^	Palmitoleic Acid (C16:1) ^2^
Larvae *A. mellifera*	Brood	[3]	n.a.	n.a.	n.a.	n.a.	12,897.9	n.a.
Pupae ^1^ *A. mellifera*	Brood	[3]	n.a.	n.a.	n.a.	n.a.	8900.6 to 10,275.7	n.a.
Prepupae *A. mellifera mellifera*	Drones	[26,28]	3.1	n.a.	72.3	n.a.	4439.6	6.6
Early (white-eyed) pupae *A. mellifera mellifera*	Drones	[26,28]	3.0	n.a.	65.6	n.d.	4578.5	7.6
Late (dark-eyed) pupae *A. mellifera mellifera*	Drones	[26,28]	2.4	n.a.	56.1	n.a.	4197.3	8.5
Larvae *A. mellifera ligustica*	Drones	[25]	n.a.	35.1	n.a.	n.a.	2346.1	n.d.
Early (white-eyed) pupae *A. mellifera ligustica*	Drones	[26]	n.d.	n.a.	47.7	6.8	4902.8	8.7
Late (dark-eyed) pupae *A. mellifera ligustica*	Drones	[26]	n.d.	n.a.	48.3	n.d.	4412.0	10.4
Pupae *A. mellifera ligustica*	Workers	[25]	n.a.	31.1	n.a.	n.a.	2632.1	n.d.
Prepupae *A. mellifera carnica*	Drones	[26,28]	2.4	n.a.	55.4	n.a.	4701.8	7.3
Early (white-eyed) pupae *A. mellifera carnica*	Drones	[26,28]	2.0	n.a.	50.8	n.d.	4771.3	7.6
Late (dark-eyed) pupae *A. mellifera carnica*	Drones	[26,28]	n.d.	n.a.	47.9	n.a.	4316.3	9.1
Prepupae *A. mellifera buckfast*	Drones	[26]	n.d.	56.4	n.d.	n.a.	4720.3	n.d.
Late (dark-eyed) pupae *A. mellifera buckfast*	Drones	[26]	n.d.	51.9	n.d.	n.a.	5104.5	n.d.

n.a. = not available; n.d. = not detected. ^1^ Range of values corresponding to pupae fed with different diets. ^2^ Palmitoleic acid and 9-hexadecenoic acid are stereoisomers (t stands for the trans version, while the cis version does not have the t); ^3^ Elaidic acid and oleic acid are stereoisomers (t stands for the trans version, while the cis version does not have the t).

**Table A5:** Polyunsaturated fatty acids of *A. mellifera* subspecies *mellifera, ligustica, carnica*, and *buckfast*.

Species/Subspecies and Developmental Stage, When Available	Honey Bee Caste	Reference	Polyunsaturated Fatty Acids (g/100 g Dry Matter)					
			Linolelaidic Acid (C18:2t) ^2^	Linoleic Acid (C18:2) ^2^	Linolenic Acid (C18:3)	Mead Acid (C20:3) ^3^	Docosadienoic Acid (C22:2)	Eicosapentaenoic Acid (C20:5)
Larvae *A. mellifera*	Brood	[3]	n.a.	421.5	730.6	n.a.	n.a.	n.a.
Pupae ^1^ *A. mellifera*	Brood	[3]	n.a.	295.4 to 401.1	420.2 to 485.3	n.a.	n.a.	n.a.
Prepupae *A. mellifera mellifera*	Drones	[26,28]	21.3	31.3	77.4	n.d.	13.0	6.5
Early (white-eyed) pupae *A. mellifera mellifera*	Drones	[26,28]	22.0	53.2	98.4	n.d.	16.8	7.4
Late (dark-eyed) pupae *A. mellifera mellifera*	Drones	[26,28]	22.2	56.8	118.7	n.d.	19.4	6.6
Early (white-eyed) pupae *A. mellifera ligustica*	Drones	[26]	n.d.	22.8	61.2	n.d.	15.2	n.d.
Late (dark-eyed) pupae *A. mellifera ligustica*	Drones	[26]	n.d.	30.7	83.2	n.d.	17.2	n.d.
Prepupae *A. mellifera carnica*	Drones	[26,28]	10.2	46.6	153.0	n.d.	14.9	3.9
Early (white-eyed) pupae *A. mellifera carnica*	Drones	[26,28]	13.3	49.0	151.9	n.d.	18.8	6.0
Late (dark-eyed) pupae *A. mellifera carnica*	Drones	[26,28]	17.3	36.3	154.1	1.8	26.2	7.3
Late (dark-eyed) pupae *A. mellifera buckfast*	Drones	[26]	n.a.	67.9	n.a.	n.a.	n.a.	n.a.

n.a. = not available; n.d. = not detected. ^1^ Range of values corresponding to pupae fed with different diets. ^2^ Linolelaidic acid and linoleic acid are stereoisomers (t stands for the trans version, while the cis version does not have the t). ^3^ Mead acid or eicosatrienoic acid.

**Table A6:** Minerals in the bee brood of *A. mellifera* subspecies *mellifera, ligustica, carnica, buckfast*, and others.

Species/Subspecies and Developmental Stage, When Available	Caste	Ref.	Mineral Content (mg/100 g Dry Matter)													
			Bromine (Br)	Calcium (Ca)	Chloride (Cl)	Gold (Au)	Iodine (I)	Iron (Fe)	Magnesium (Mg)	Manganese (Mn)	Phosphorus (P)	Potassium (K)	Selenium (Se)	Sodium(Na)	Strontium (Sr)	Zirconium (Zr)
Prepupae *A. mellifera mellifera*	Drones	[26,28]	n.a.	39.3	n.a.	n.a.	n.a.	4.7	70.2	n.a.	673.5	1079.9	n.a.	8.1	n.a.	n.a.
Early (white-eyed) pupae *A. mellifera mellifera*	Drones	[26,28]	n.a.	40.1	n.a.	n.a.	n.a.	5.2	75.3	n.a.	731.3	1205.2	n.a.	8.7	n.a.	n.a.
Late (dark-eyed) pupae *A. mellifera mellifera*	Drones	[26,28]	n.a.	43.3	n.a.	n.a.	n.a.	5.7	85.8	n.a.	812.3	1341.6	n.a.	9.9	n.a.	n.a.
Larvae *A. mellifera ligustica*	Workers	[25]	n.a.	331.6	n.a.	n.a.	n.a.	52.0	691.4	4.7	3056.6	7312.1	n.a.	232.0	n.a.	n.a.
Early (white-eyed) pupae *A. mellifera ligustica*	Drones	[26]	n.a.	43.7	n.a.	n.a.	n.a.	4.9	82.9	n.a.	774.0	544.6	n.a.	7.3	n.a.	n.a.
Late (dark-eyed) pupae *A. mellifera ligustica*	Drones	[26]	n.a.	49.3	n.a.	n.a.	n.a.	5.7	95.0	n.a.	892.4	643.1	n.a.	8.5	n.a.	n.a.
Pupae *A. mellifera ligustica*	Workers	[25]	n.a.	97.0	n.a.	n.a.	n.a.	15.3	193.9	0.7	900.0	2207.3	n.a.	60.8	n.a.	n.a.
Prepupae *A. mellifera carnica*	Drones	[26,28]	n.a.	34.0	n.a.	n.a.	n.a.	5.6	65.9	n.a.	651.7	1048.9	n.a.	7.8	n.a.	n.a.
Early (white-eyed) pupae *A. mellifera carnica*	Drones	[26,28]	n.a.	37.9	n.a.	n.a.	n.a.	5.7	74.3	n.a.	734.7	1219.8	n.a.	7.0	n.a.	n.a.
Late (dark-eyed) pupae *A. mellifera carnica*	Drones	[26,28]	n.a.	46.1	n.a.	n.a.	n.a.	6.1	88.4	n.a.	869.2	1401.2	n.a.	10.3	n.a.	n.a.
Prepupae *A. mellifera buckfast*	Drones	[26]	n.a.	34.2	n.a.	n.a.	n.a.	5.6	68.1	n.a.	686.9	891.1	n.a.	30.1	n.a.	n.a.
Late (dark-eyed) pupae *A. mellifera buckfast*	Drones	[26]	n.a.	38.7	n.a.	n.a.	n.a.	6.0	81.9	n.a.	802.6	1102.0	n.a.	38.0	n.a.	n.a.
Brood (subspecies not indicated)	Brood	[24]	n.a.	59.5	375.0	n.a.	<0.04	5.6	90.9	0.3	771.6	1159.5	0.026	55.2	n.a.	n.a.
Larvae and pupae (subspecies not indicated)	Drones	[40]	0.195	133.6	n.d.	0.12	n.a.	6.087	n.a.	0	n.a.	n.a.	n.a.	n.a.	0.313	0.167

n.a. = not available; n.d. = not detected.

**Table A7:** Heavy metals in the bee brood of *A. mellifera* subspecies *mellifera, ligustica, carnica, buckfast*, and others.

Species/Subspecies and Developmental Stage, When Available	Caste	Ref.	Heavy Metal Content (mg/100 g Dry Matter)								
			Bismuth (Bi)	Cadmium (Cd)	Chromium (Cr)	Copper (Cu)	Lead (Pb)	Mercury (Hg)	Molybdenum (Mo)	Silver (Ag)	Zinc (Zn)
Prepupae *A. mellifera mellifera*	Drones	[26,28]	n.a.	n.a.	n.a.	1.5	n.a.	n.a.	n.a.	n.a.	4.4
Early (white-eyed) pupae *A. mellifera mellifera*	Drones	[26,28]	n.a.	n.a.	n.a.	1.6	n.a.	n.a.	n.a.	n.a.	4.9
Late (dark-eyed) pupae *A. mellifera mellifera*	Drones	[26,28]	n.a.	n.a.	n.a.	1.9	n.a.	n.a.	n.a.	n.a.	5.5
Larvae *A. mellifera ligustica*	Workers	[25]	n.a.	n.a.	n.a.	14.1	n.a.	n.a.	n.a.	n.a.	45.3
Early (white-eyed) pupae *A. mellifera ligustica*	Drones	[26]	n.a.	n.a.	n.a.	1.8	n.a.	n.a.	n.a.	n.a.	5.3
Late (dark-eyed) pupae *A. mellifera ligustica*	Drones	[26]	n.a.	n.a.	n.a.	1.9	n.a.	n.a.	n.a.	n.a.	5.9
Pupae *A. mellifera ligustica*	Workers	[25]	n.a.	n.a.	n.a.	3.7	n.a.	n.a.	n.a.	n.a.	11.7
Prepupae *A. mellifera carnica*	Drones	[26,28]	n.a.	n.a.	n.a.	1.6	n.a.	n.a.	n.a.	n.a.	4.8
Early (white-eyed) pupae *A. mellifera carnica*	Drones	[26,28]	n.a.	n.a.	n.a.	1.8	n.a.	n.a.	n.a.	n.a.	5.3
Late (dark-eyed) pupae *A. mellifera carnica*	Drones	[26,28]	n.a.	n.a.	n.a.	2.0	n.a.	n.a.	n.a.	n.a.	6.0
Prepupae *A. mellifera buckfast*	Drones	[26]	n.a.	n.a.	n.a.	0.1	n.a.	n.a.	n.a.	n.a.	5.1
Late (dark-eyed) pupae *A. mellifera buckfast*	Drones	[26]	n.a.	n.a.	n.a.	0.4	n.a.	n.a.	n.a.	n.a.	6.0
Brood (subspecies not indicated)	Brood	[24]	n.a.	n.a.	n.a.	1.7	n.a.	n.a.	n.a.	n.a.	6.9
Larvae and pupae (subspecies not indicated)	Drones	[40]	0.387	n.d.	n.d.	5.483	0.21	n.d.	0.45	8.27	n.a.

n.a. = not available; n.d. = not detected.
